# The Impact of Alzheimer’s Disease and Related Dementias on Dental Care Utilization and Costs in Older Adults

**DOI:** 10.1093/geroni/igaa057.2903

**Published:** 2020-12-16

**Authors:** Sam Li, Isaac Donkor, Liang Hong, Kevin Lu, Bei Wu

**Affiliations:** 1 University of Tennessee Health Science Center, Memphis, Tennessee, United States; 2 Vanderbilt University, Nashville, Tennessee, United States; 3 University of South Carolina, Columbia, South Carolina, United States; 4 New York University, New York, New York, United States

## Abstract

There is limited information on the impact of cognition function on dental care utilization and costs. This study used the Medicare current beneficiaries survey in 2016 and included 4,268 participants 65+. Dental care utilization and costs were measured by self-report and included preventive and treatment events. Negative binomial regression and generalized linear regression were used to examine the impact of Alzheimer’s disease (AD) and related dementia (RD) on dental care utilization and costs. We found that AD was not associated with dental care utilization, but RD was associated with a lower number of total treatment dental care visits (IRR: 0.60; 95% CI: 0.37~0.98). RD was not associated with dental care costs, but AD was associated with higher total dental care costs (estimate: 1.08; 95% CI: 0.14~2.01) and higher out-of-pocket costs (estimate: 1.25; 95% CI: 0.17~2.32). AD and RD had different impacts on different types of dental care utilization and costs. Part of a symposium sponsored by the Oral Health Interest Group.

